# Weifuchun alleviates MNNG-induced chronic atrophic gastritis by improving the gastric and intestinal microbiota homeostasis

**DOI:** 10.1371/journal.pone.0333375

**Published:** 2025-11-24

**Authors:** Yingying Chen, Ping Feng, Xiaoqin Lou, Haiying Wang, Yanlan Hong, Jiaojiao Yang, Shenbao Wu

**Affiliations:** 1 Department of Gastroenterology, Yiwu Central Hospital of Wenzhou Medical University, Yiwu, China; 2 Department of Gastroenterology, Sir Run Run Shaw Hospital of Zhejiang University School of Medicine, Hangzhou, China; Hong Kong Baptist University, HONG KONG

## Abstract

**Purpose:**

Chronic atrophic gastritis (CAG) is a prevalent condition that can undergo precancerous lesions of gastric cancer (PLGC) and thus progression to gastric cancer. Weifuchun (WFC), a traditional Chinese herbal compound, is employed in CAG treatment and microbial homeostasis modulation. We aimed to explore the role of WFC in CAG from the perspective of gastrointestinal microbiota.

**Methods:**

PLGC rats were developed by N-methyl-N’-nitro-N-nitrosoguanidine treatment. Then, various doses of WFC were administered to the rats and WFC compositions were analyzed. The pathological changes and inflammatory markers of the rats were assessed. Key proteins in the interleukin (IL)-6/Janus Kinase 1/Signal Transducer and Activator of Transcription 3 (JAK1/STAT3) pathway were evaluated, alongside fecal microbial analysis through 16S ribosomal DNA sequencing.

**Results:**

Most compounds identified from WFC were nucleosides, flavones, organic acids, phenylpropanoids and other components. WFC treatment alleviated intestinal metaplasia and dysplasia caused by atrophic gastritis in PLGC rats. WFC also reduced inflammatory marker levels, and inhibited the IL-6/JAK1/STAT3 pathway for PLGC rats. The higher the dose of WFC was administrated, the better the effects on the aforementioned results. Simultaneously, WFC improved the CAG gastrointestinal microbiota homeostasis. Interestingly, WFC increased *Coprocuccus* abundance, which was significantly correlated with proliferating cell nuclear antigen and IL-6 levels.

**Conclusions:**

WFC alleviated PLGC by regulating the balance of gastrointestinal microbiota and influencing the IL-6/JAK1/STAT3 pathway, revealing the potential connection between gastrointestinal microbiota and CAG progression. This study is the first to link gastrointestinal microbiota homeostasis and IL-6/JAK1/STAT3 pathway, providing novel insights into WFC’s multi-targeted therapeutic mechanism on CAG.

## Introduction

Chronic atrophic gastritis (CAG) is a recognized precancerous lesion for gastric cancer (PLGC), which is the fifth most common cancer worldwide and the fifth leading cause of cancer-related deaths [1]. CAG is characterized by inflammatory cell infiltration and glandular atrophy in the gastric mucosa [2]. CAG may progress to gastric cancer following the development of intestinal metaplasia and dysplasia in the gastric mucosa. The presence of intestinal metaplasia and dysplasia significantly elevates the risk of gastric cancer occurrence [3,4]. CAG primarily includes two types: *Helicobacter pylori*-associated CAG (multifocal) and autoimmune CAG (body-limited) [5]. Although *Helicobacter pylori* is a major contributor to CAG, the overall etiology remains poorly understood. The Operative Link on Gastritis Assessment system is commonly used for evaluation, but many patients are still diagnosed at advanced stages, since effective treatment options are limited [2]. Considering that gastric mucosal intestinal metaplasia and dysplasia are significant risk factors for gastric cancer, it is essential to implement effective interventions for PLGC prior to the onset of malignancy. Thus, there is an urgent need for the development of improved diagnostic and therapeutic strategies for CAG.

Chronic inflammation and inflammatory cells are pivotal in gastrointestinal diseases, particularly in the progression from CAG to PLGC and ultimately to gastric cancer [6,7]. Interleukins (IL) play essential roles in CAG development and epithelial cell dynamics. IL-6 is a pro-inflammatory cytokine that is often used to assess the severity of gastric mucosal loss [8]. Gastric mucosal lesions can lead to abnormal increases in IL-6 levels [9]. High-throughput sequencing has also showed that IL-6 is highly expressed in the gastric tissue of PLGC mice compared with normal gastric tissue [10]. Studies have shown that down-regulating IL-6, IL-17RA and tumor necrosis factor-alpha (TNF-α) levels can effectively improve the pathological changes of gastric mucosa tissue and hinder the development of PLGC into gastric cancer [11]. An increasing number of studies have indicated that the composition and changes of the gastrointestinal microbiota are associated with inflammation and cancer [12–14]. The dynamic relationship between gastric and intestinal microbiota [15], marked by microbial migration, metabolite exchange, and co-regulation of the host immune system, underscores their integral roles in gastrointestinal health, with gastric microbiota influencing intestinal composition via food residues and gut microbiota affecting gastric function through the microbiota-gut-brain axis [16]. Additionally, bacteria producing lipopolysaccharides in cancer patients may activate the IL-6/Janus Kinase 1 (JAK1)/Signal Transducer and Activator of Transcription 3 (STAT3) signaling pathway [17], indicating that the imbalance of gastric microbiota has potential roles in the formation of CAG, PLGC, and gastric cancer, and the IL-6/JAK1/STAT3 signaling pathway may be involved. In addition, the gastric microbiota predominantly comprises *Helicobacter pylori* and non-HP bacteria [18,19]. Studies have demonstrated a notable increase in *Lactobacillus* and *Bifidobacterium* in PLGC model rats, correlating with serum inflammatory factor changes [12]. This indicates a link between changes in the gastrointestinal microbiota environment and inflammatory factors and gastric microbiota [20].

Weifuchun (WFC), a traditional Chinese herbal compound, comprised mainly of Renshen (*Red ginseng*), Xiangchacai (*Isodon amethystoides*), and Zhike (*Fructus Aurantii*), is widely used in clinical practice to treat PLGC, postoperative gastric cancer, and chronic non-atrophic gastritis [21]. Research has indicated that WFC can inhibit TNF-α and IL-6 levels in PLGC rat models while modulating key genes like Toll-like receptor 14 (TLR14) and CD6. Additionally, cellular studies have revealed that WFC mitigates PLGC by downregulating mRNA expressions of TNF-α, IL-6, IL-1, and STAT3 [22,23]. Studies have shown that WFC capsules can fight PLGC through specific immunoregulatory effects and Toll-like receptor signaling pathways, especially by inhibiting inflammatory factors [24]. While recent studies have indicated that WFC can enhance gut microbiota and support homeostasis in the intestinal system, the specific regulatory effects of WFC on gastric microbiota remain unclear [25,26]. The regulatory influence of WFC on gastric and intestinal microbiota appears to be a crucial mechanism in ameliorating gastritis, while the limited scientific evidence concerning the interactions between gastric mucosal and gut microbiota in conditions like PLGC underscores the necessity for further research.

Therefore, we used WFC-treated PLGC rats and investigated the pathological changes in gastric mucosal tissues, inflammatory factors, the JAK1/STAT3 signaling pathway, as well as using 16S rRNA sequencing and correlation analysis to investigate the microbial community of the gastric contents and feces, which aims to investigate the effects and potential mechanisms of WFC treatment on PLGC.

## Materials and methods

### Animal and drug information

A total of 34 Specific pathogen free (SPF) Sprague-Dawley (SD) male rats, aged 4–6 weeks and weighing between 180 and 200 g, were obtained from Shanghai SLAC Laboratory Animal Co., Ltd. (Certificate No. SCXK (Hu) 2017−0005; China) and were maintained under SPF conditions with free access to regular food and water [27]. All animal experiments were approved by the Laboratory Animal Management and Ethics Committee of Hangzhou Hunter Testing Biotechnology Co., Ltd. [ethics approval number: IACUC/HTYJ-8201–19 and approval date: February 28, 2024] and strictly adhered to the guidelines for the care and use of laboratory animals. In addition, in this study, we did everything we could to lessen animals pain and suffering.

N-methyl-N’-nitro-N-nitrosoguanidine (MNNG, D1218D) was procured from MeilunBio Co., Ltd. in Dalian, China, and WFC capsules (23111120) were purchased from the Hangzhou Huqingyutang Group Co., Ltd (Hangzhou, China). The preparation of a 0.25 g/mL WFC solution was conducted by dissolving 2.5 g of WFC powder in 10 mL of double-distilled water. The mixture was subjected to a water bath at 37°C for 30 min to facilitate dissolution, followed by sonicating using a VC130PB ultrasonic processor (500 w, 40 KHz, 50 °C, Sonics & Materials Inc., USA) for 1 h to ensure thorough integration of the components. Subsequently, the solution underwent centrifugation at room temperature (7,000 × g, 2 min) to separate any undissolved materials. The resulting supernatant was then filtered through a 0.22 μm filter to obtain a clear solution [22]. This experiment was conducted at the Research Center for Huante Biology Science and Technology Co., Ltd.

### Ultra-high performance liquid chromatography with quadrupole time-of-flight mass spectrometry (UPLC-Q-TOF-MS) analysis

To analyze the compositions of WFC, 200 μL of WFC solution (0.25 g/mL) was taken from −80°C and subsequently diluted with a precooled pure methanol/pure acetonitrile mixture in a 1:1 volume ratio. The obtained solution was vortexed, followed by protein precipitation at −20°C for 2 h. Then, centrifugation was performed to the sample for obtaining the supernatant, and the obtained supernatant was then concentrated to dryness under vacuum conditions. The dried sample was reconstituted in a 0.1% (v/v) formaldehyde solution and analyzed utilizing a UPLC-Q-TOF-MS. Detailed information regarding the chromatographic and mass spectrum parameters could be found in S1 Table. In this study, data collection and analysis were conducted using SCIEX OS software.

### MNNG-induced PLGC model

After a period of three days dedicated to adaptive feeding, the rats were randomly divided into the normal control group (NC group, *n* = 8) and the model group (*n* = 26). After that, all rats, with the exception of those in the NC group, were given 150 μg/mL MNNG aqueous solution for drinking (protected from light) and received the intragastric administration of MNNG at a dosage of 10 mL/kg (170 μg/mL) every day [28]. At the same time, the NC rats were drink pure water and gavaged with an equal volume of pure water every day. The model rats were specifically fed every two days, followed by a fasting period of one day [22]. Additionally, these model rats received a gavage of 1 mL/100 g of 40% ethanol twice weekly over the course of ten weeks [29]. Histopathological analysis was conducted on two rats randomly selected from the NC and model groups to confirm the efficacy of the model establishment via Hematoxylin-eosin (HE) staining.

### Grouping and sampling

Following the successful determination of the model at the ten-week mark, the model rats were randomly categorized into four distinct groups: the model group, and the high (1.2 g/kg/day), medium (0.6 g/kg/day), and low (0.3 g/kg/day) doses of the WFC treatment groups (*n = 6*) [22]. The medication treatment lasted for eight weeks [22]. During these eight weeks of medication treatment, the model group and WFC treatment groups continued to receive MNNG administration until the end of the treatment period [30].

Immediately after the rats were euthanized with carbon dioxide, abdominal aortic blood was collected, and gastric tissue was isolated for use in experiments. Blood was immediately centrifuged (3000 rpm, 10 min) to collect serum for ELISA. After visual observation of gastric mucosa morphology, gastric mucosa tissue was taken and part of the gastric mucosa was fixed in 4% paraformaldehyde, and another part of the gastric mucosa was used for Western blot.

### HE staining

As reported by the previous report, dehydrated and embedded gastric mucosa was fixed for 24 h, and used a slicer (Leica Microsystems (Shanghai) Co., Ltd., RM2235) to slice the wax block into thin slices with a thickness of about 4 μM [31]. The nucleus was stained with hematoxylin staining solution for 5 min (Sigma, H3136). Then, the samples were rinsed with running water to remove excess hematoxylin dye. Differentiation was performed using 1% ethanol hydrochloride to optimize staining of the cell nucleus. Rinse with running water again, and then use 40% ammonia or warm water to turn blue cell nuclei. The cytoplasm was stained with an eosin staining solution for 5 min (Sigma, E4009). The stained sections were dehydrated through an absolute ethanol series and a xylene series, and the sections were made transparent. Seal sections with neutral gum (Sinopharm Chemical Reagent Co., Ltd., 10004160) to prevent fading and facilitate microscopic observation using microscopy (Nikon Corporation, Nikon Eclipse Ci-L), then perform image acquisition and analysis. Experienced pathological researchers evaluated histopathology in a blind manner.

### Enzyme-linked immunosorbent assay (ELISA)

Rat aortic blood was collected to isolate the serum supernatant. As reported by the previous report, the levels of TNF-α, IL-6, IL-1β, IL-8, pepsinogen I/pepsinogen II (PGI/PGII), and gastrin (Gas) were quantified using a rat ELISA detection kit [32]. Each specific antibody or antigen was coated on enzyme-labeled plates, and incubated overnight at 4°C, followed by blocking with a protein solution, addition of samples, incubation at 37°C, washing, and development with two developer solutions, with the absorbance measured at 450 nm using a microplate reader (CMaxPlus, MD). This experiment was conducted in six replicates.

The TNF-α (RX302058R), IL-6 (RX302856R), IL-1β (RX302869R), IL-8 (RX2D302216), and Gas (RX302271R) assay kits were purchased from RUIXIN BIOTECH. The PGI (CB10829-Ra) and PGII (CB10830-Ra) assay kits were purchased from COIBO BIO.

### Western blot

As described in a previous study, Western blot was applied to detect the expressions of IL-6/JAK1/STAT3 signaling pathway-related protein in rat gastric mucosa [33]. In short, 100 mg of rat gastric mucosa was prepared, carefully cut and placed into a tube. 1 mL of pre-cooled RIPA lysis solution (Beytome, P0013B) was added and homogenized three times in a tissue homogenizer (Pro200, PRO Scientific) in a 20-second cycle. After standing on ice for 30 min, transfer to a centrifuge tube and centrifuge at 12,000 × g at 4°C for 5 min. The supernatant was collected and the total protein was measured using a BCA kit (Beytome, pc0020). The sample adding buffer was denatured in a boiling water bath for 5 min. After cooling at room temperature, it was stored at −20°C. Proteins were separated by SDS-PAGE (Beyotime, P0012A) and transferred to a PVDF membrane (GE Healthcare Life, 10600023), and 5% skimmed milk TBST blocking reduced non-specific binding. Specific primary and secondary antibodies were incubated in turn to enhance the signal, and ECL luminescence detected the protein to achieve accurate visualization and perform grayscale analysis on protein bands using Image J software. The antibody information and dilution ratio were detailed in Table 1.

**Table 1:** Antibody information used for Western blot.

Antibody	Brand	No.	Dilution ratio
**IL-6 Antibody**	CST	12912S	1:1000
**p-JAK1 Antibody**	Affinity	AF2012	1:1000
**JAK1 Antibody**	Affinity	AF5012	1:1000
**p-STAT3 Antibody**	Affinity	AF3293	1:1000
**STAT3 Antibody**	Affinity	AF6294	1:1000
**Anti-rabbit IgG, HRP-linked Antibody**	CST	7074	1:6000
**GAPDH Antibody**	Proteintech	10494-1-AP	1:10000

### Immunohistochemistry (IHC)

As reported by the previous report, IHC was performed to measure PCNA expression in rat gastric mucosa [34]. In short, dehydrated and embedded gastric mucosa was fixed for 24 h, and slice the wax block into thin slices with a thickness of about 4 μM. Elimination of endogenous peroxidase activity with a 3% hydrogen peroxide solution reduces background staining. The non-specific binding site was blocked with 5% BSA. Incubate overnight with proliferating cell nuclear antigen (PCNA) antibody (Proteintech, 10205–2-AP, 1:200) at 4°C. Incubation with Goat-anti-rabbit IgG H&L (HRP) preadsorbed secondary antibody (Abcam, ab97080, 1:5000) at 37°C for 30 min. Use DAB solution for color development. Cell nuclei were counterstained with dyes such as hematoxylin to provide contrasting background. Sections were dehydrated through a series of alcohol solutions with increasing concentrations, then cleared through xylene, and sealed with resin. Under microscopic examination, the presence of yellow or brownish-yellow pigmentation in the cytoplasm or membrane signaled a positive expression.

Images were acquired and processed for semi-quantitative analysis. Each sample’s integrated optical density (IOD) and area value (sample area under 200 × magnification) were measured, with the IOD/Area ratio serving as a key indicator for semi-quantitative assessment.

### 16S rRNA sequencing

Gastric contents [35] and fecal samples [36] from rats in the NC group (*n* = 6), PLGC group (*n* = 6), and PLGC+H-WFC group (*n* = 6) were collected, immediately frozen and stored at −80 °C. Then, these samples were aseptically transferred into sterile DNase/RNase-free 2.0 mL tubes for the extraction of the genomic DNA from the existing microorganisms. Universal primers targeting the 16S rRNA gene were employed for polymerase chain reaction (PCR) amplification to produce a sufficient quantity of DNA fragments suitable for sequencing. The V3–V4 hypervariable regions of the bacteria 16S rRNA gene were amplified with primers 338F (5′-ACTCCTACGGGAGGCAGCAG-3′) and 806R (5′-GGACTACHVGGGTWTCTAAT-3′) by a thermocycler PCR system. Impurities and non-specific amplification products from the PCR reactions were removed using magnetic beads from the Vazyme VAHTSTM DNA Clean Beams kit. The purified PCR product was then ligated to a sequencing vector to construct a sequencing library using the Illumina TruSeq Nano DNA LT Library Prep Kit. The quality and concentration of the resulting library were assessed through fluorescence quantification with the Quant IT PicoGreen dsDNA Assay Kit and gel electrophoresis. Subsequently, leading sequencing platforms such as Illumina were utilized to obtain extensive short read sequences.

The raw sequencing data were comprehensively analyzed using QIIME2 [37] software, including quality control, sequence concatenation, noise removal, error correction, and operational taxonomic unit (OTU) clustering. Compare the processed sequence with the Silva database [38] (http://www.arb-silva.de) to determine the classification of microorganisms. In addition, diversity analysis, community structure analysis, and functional prediction were conducted to elucidate the composition and function of microbial communities [39]. In order to evaluate the alpha diversity of microbial communities, this process characterizes richness using Chao1 and Observed species indices, diversity using Shannon and Simpson indices, evolutionary diversity using Faith’s PD index, evenness using Pielou’s evenness index, and coverage using Good’s coverage index. To evaluate beta diversity distance, this process characterizes it using Jaccard distance, Bray Curtis distance, unweighted UniFrac distance, and weighted UniFrac distance. Cluster analysis was conducted using the unweighted pair group method with arithmetic means (UPGMA). Key taxa were determined based on an FDR threshold of 0.05. Linear discriminant analysis (LDA) effect size (LEfSe) was used to identify significant differences in relative abundances of gastric contents and intestinal microbiota among groups. The groups with LDA scores > 2 and *P < 0.05* were considered significant.

### Statistical analysis

The data of the study were analyzed using SPSS 20.0, with the values presented as average values ± standard deviation (SD). For comparisons involving multiple groups, a one-way analysis of variance followed by Tukey tests was conducted. Additionally, the Kruskal-Wallis H test was utilized when the data didn’t meet the criteria for normal distribution, and the Dunnett T3 test was used for data with unequal variances. *P < 0.05* was deemed statistically significant. All quantitative experiments were conducted in triplicate to ensure the reliability and reproducibility of the results.

Post-hoc Test: A one-way ANOVA was performed, followed by Tukey’s HSD post-hoc test for pairwise comparisons where the main effect was significant.

FDR Correction: For multiple comparison correction across, the FDR was controlled using the Benjamini-Hochberg method. An FDR-adjusted p-value (q-value) of < 0.05 was considered statistically significant.

## Results

### Component analysis of WFC

**Fig 1:**
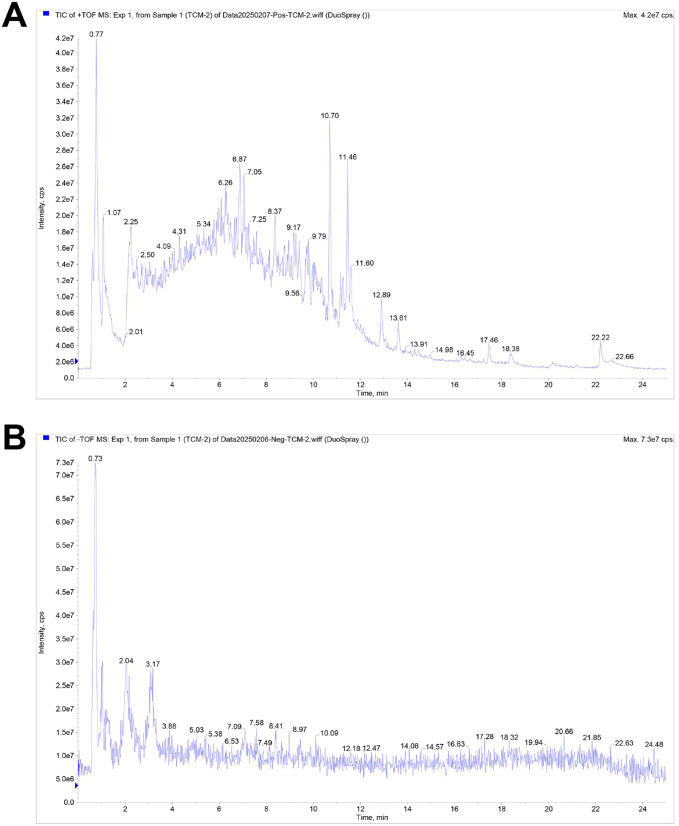
Composition analysis of WFC by UPLC-Q-TOF-MS. Total ion flow chromatogram of WFC in (A) positive ion mode and (B) negative ion mode.

The chemical compositions of WFC were analyzed using UPLC-Q/TOF-MS. The total ion flow diagram of WFC was presented in Fig 1. A total of 78 components were identified in the positive ion mode, and 77 components were found in the negative ion mode. These components included nucleosides, flavones, organic acids, phenylpropanoids and other components, as detailed in S2 and S3 Tables.

### MNNG-induced PLGC model in SD rats

**Fig 2:**
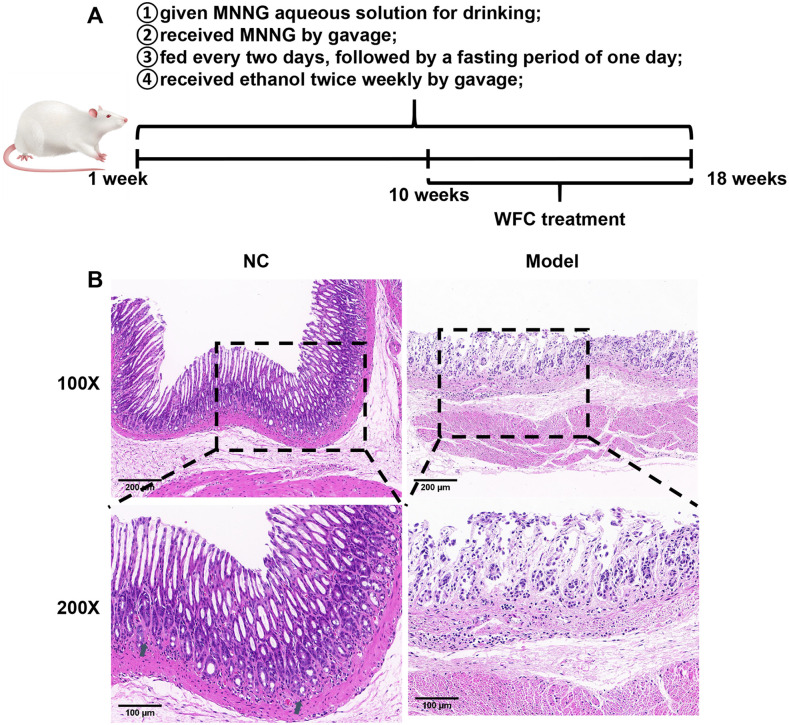
MNNG-induced chronic atrophic gastritis model in SD rats. **(A)** The PLGC SD rat model was established by MNNG induction. **(B)** HE staining of gastric mucosa in MNNG-induced PLGC SD rats (Magnification 100 × , scale bar 200 μm; magnification 200 × , scale bar 100 μm).

To investigate the therapeutic effect of WFC on CAG, a PLGC rat model was established by MNNG induction, and the successful modeling was confirmed by HE staining after a 10-week model period (Fig 2A). As shown in Fig 2B, the gastric mucosal tissue of rats in the NC group showed normal morphology. The PLGC group of rats exhibited pathological conditions characterized by gastric mucosal intestinal metaplasia and atypical hyperplasia. Notably, there was a significant loss of gastric mucosal epithelial cells, which were largely replaced by intestinal-type epithelial cells. Furthermore, there was pronounced atrophy of the glands, a reduction in structural integrity, and a marked infiltration of inflammatory cells.

### WFC ameliorates gastric atrophy in the MNNG-induced PLGC rat model

**Fig 3:**
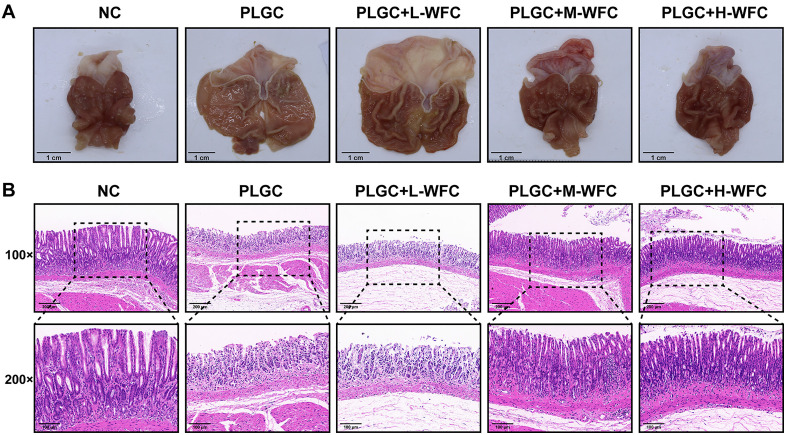
WFC ameliorates gastric atrophy in the MNNG-induced PLGC rat model. **(A)** Observation of gastric mucosal morphology in rats. **(B)** Histopathological changes of gastric mucosa in rats (Magnification 100 × , scale bar 200 μm; magnification 200 × , scale bar 100 μm).

After an eight-week drug intervention, we examined tissue morphology and pathological conditions. Compared to the PLGC group, rats receiving WFC treatment exhibited notable improvements in gastric mucosal morphology, characterized by a gradual reddening of the gastric tissue, thickening of the gastric wall, and deeper folds. Furthermore, the enhancements in gastric mucosal morphology were proportionate to the concentration of WFC administered (Fig 3A). HE staining indicated that the gastric mucosal tissue in the WFC treatment group of rats exhibited varying degrees of improvement in pathological conditions such as intestinal metaplasia and atypical hyperplasia. Specifically, there was a reduction in gastrointestinal epithelial cells, normalization of gastric mucosal cell morphology, higher levels of cellular differentiation, and decreased infiltration of lymphocytes and plasma cells in the submucosal layer. Notably, higher doses of WFC corresponded to milder severity of lesions (Fig 3B).

### Immunohistochemical determination of PCNA expression in rat gastric tissue

**Fig 4:**
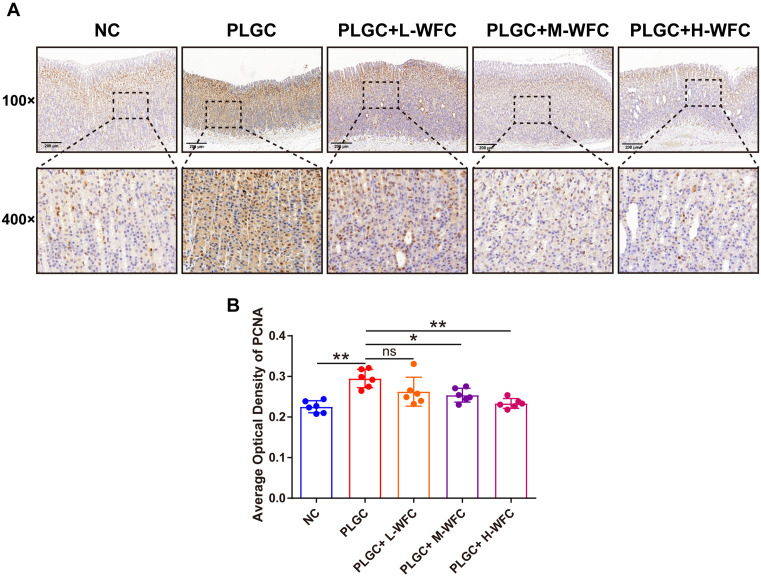
Immunohistochemical determination of PCNA expression in rat gastric tissue. **(A)**The expression level of PCNA in gastric mucosal tissue of rats in each group (Magnification 100 × , scale bar 200 μm; magnification 400 × , scale bar 50 μm); **(B)** The expression level of PCNA in gastric mucosal tissue of rats in each group. Mean±standard deviation, *n* = 6. ^*^P < 0.05, ^**^P < 0.01.

The pathological alterations associated with PLGC are closely linked to the proliferation of gastric mucosa, prompting our investigation into the expression of PCNA within this tissue through immunohistochemical analysis. As illustrated in Fig 4A, the NC group exhibited a limited presence of PCNA-expressing cells, predominantly localized to the proliferative zone, characterized by a regular distribution and faint staining. Conversely, in untreated rats with PLGC, there was a marked increase in the number of PCNA-positive cells, which were widely scattered and displayed darker staining across multiple layers. Notably, rats in the PLGC+H-WFC group demonstrated expression patterns and distributions akin to those observed in the NC group, with fewer PCNA-expressing cells primarily situated in the proliferation zone. Statistical assessment of the immunohistochemistry data further confirmed that relative to the NC group, the average optical density (AOD) values of PCNA in the gastric mucosal tissues of the PLGC group was increased by 30.84% (*P < 0.01*). Meanwhile, the AOD values noted in the PLGC+M-WFC and PLGC+H-WFC groups were significantly diminished by 13.84% and 20.78% when compared to the PLGC group, respectively (Fig 4B, *P < 0.05, P < 0.01*).

### Detection of inflammation-related cytokines and gastric state-related biochemical indicators in rat serum

**Fig 5:**
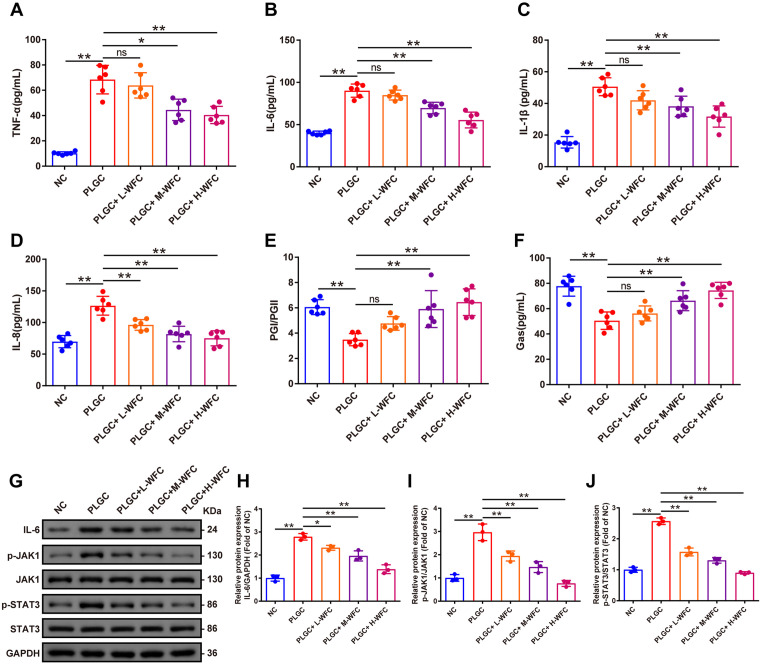
Detection of inflammatory-related cytokines and gastric mucosal proteins in rat serum. **(A-F)** Levels of TNF-α, IL-6, IL-1β, IL-8, PGI/PGII, and Gas in rat serum; **(G)** Band diagram of IL-6, p-JAK1, JAK1, p-STAT3, and STAT3 protein expression in gastric mucosal tissue; **(H-J)** Expression of IL-6, p-JAK1, and p-STAT3 proteins in gastric mucosal tissue. Mean±standard deviation, *n* = 6. ^*^P < 0.05, ^**^P < 0.01.

To further investigate the systemic inflammatory state in rat models, we employed ELISA to quantify the concentrations of inflammatory cytokines present in the serum of the rats. The data illustrated in Fig 5A-5F clearly demonstrated a marked elevation in serum levels of pro-inflammatory factors, including TNF-α, IL-6, IL-1β, and IL-8, among rats in the PLGC group when compared to the NC group (TNF-α, IL-6, IL-1β, and IL-8 increased by 5.71 fold, 1.25 fold, 2.28 fold and 0.81 fold, respectively, *P < 0.01*). Conversely, indicators associated with gastric health, such as the ratios of PGI/PGII and Gas levels, exhibited a significant decline in the same cohort (PGI/PGII and Gas levels were decreased by 42.43% and 35.05%, respectively, *P < 0.01*). Moreover, a comparative analysis revealed that rats receiving WFC treatment, especially the PLGC+M-WFC and PLGC+H-WFC groups, exhibited a notable decrease in serum levels of TNF-α, IL-6, IL-1β, and IL-8, accompanied by a corresponding increase in PGI/PGII and Gas levels. Specifically, relative to the PLGC group, the PLGC+M-WFC group decreased TNF-α, IL-6, IL-1β, and IL-8 levels by 35.00%, 22.75%, 24.58% and 35.32%, respectively (*P < 0.05, P < 0.01*); the PLGC+H-WFC group decreased TNF-α, IL-6, IL-1β, and IL-8 levels by 40.89%, 38.57%, 37.34% and 40.70%, respectively (*P < 0.01*). Furthermore, relative to the PLGC group, the PLGC+M-WFC group increased PGI/PGII and Gas levels by 69.21% and 31.22%, respectively (*P < 0.01*); the PLGC+H-WFC group increased PGI/PGII and Gas levels by 84.86% and 47.30%, respectively (*P < 0.01*), which suggested a potential therapeutic effect of WFC on inflammation and gastric status. Specifically, relative to the PLGC group, PLGC+L-WFC group.

### Impact of WFC on the IL-6/JAK1/STAT3 signaling pathway in gastric mucosa

Furthermore, to elucidate the mechanism through which WFC exerts its influence, we evaluated the protein expression levels in the gastric mucosa concerning the IL-6/JAK1/STAT3 signaling pathway. The results depicted in Fig 5G-5J revealed that relative to the NC group, IL-6 expression, JAK1 phosphorylation and STAT3 phosphorylation increased by 1.79 fold, 1.96 fold and 1.57 fold, respectively (*P < 0.01*). Notably, after treatment with WFC, there were substantial reductions in IL-6 expression, JAK1 phosphorylation and STAT3 phosphorylation (*P < 0.01*). Specifically, relative to the PLGC group, PLGC+L-WFC group decreased IL-6 expression, JAK1 phosphorylation and STAT3 phosphorylation by 17.03%, 34.42% and 38.68%; PLGC+M-WFC group decreased IL-6 expression, JAK1 phosphorylation and STAT3 phosphorylation by 29.68%, 50.44% and 49.23%; PLGC+H-WFC group decreased IL-6 expression, JAK1 phosphorylation and STAT3 phosphorylation by 50.30%, 74.20% and 64.93% (*P < 0.05, P < 0.01*), which indicated a promising avenue for therapeutic intervention in inflammatory gastric disorders.

### 16S rRNA sequencing analysis of rat gastric contents

**Fig 6:**
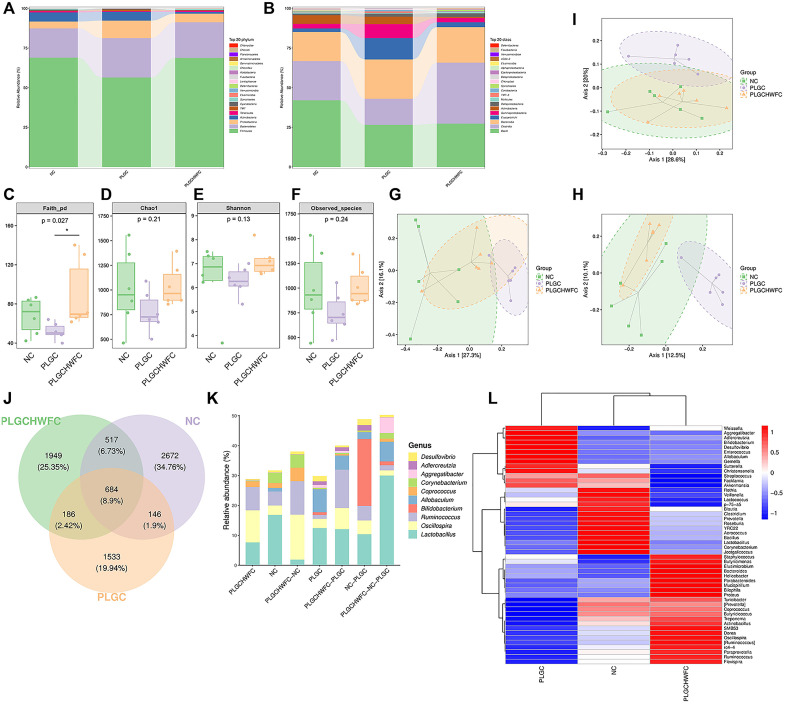
16S rRNA sequencing of gastric contents. (A) Species enrichment at the phylum level; (B) Species enrichment at the class level; (C-F) Analysis of microbial α-diversity index; (G-I) Analysis of microbial β-diversity index. (J) Venn diagram of sample ASV/OUT; (K) ASV/OTU abundance histograms in different regions of the Venn diagram at the genus level; (L) Heat map of species composition at the genus level with species clustering. *n* = 6. ^*^P < 0.05.

To investigate the specific mechanism of WFC in treating PLGC, we conducted deep sequencing of the 16S rRNA genes in gastric contents from SD rats. The dominant bacterial species in the stomach of each group of rats were analyzed. The top three species in relative abundance at the phylum level were *Firmicutes*, *Bacteroidetes* and *Proteobacteria* (Fig 6A), and the top three species in relative abundance at the class level were *Bacilli*, *Clostridia* and *Bacteroidia* (Fig 6B). Our analysis of microbial α diversity revealed a significant difference in the phylogenetic diversity of microbiota between the PLGC group and the PLGC+H-WFC group; however, no notable differences were observed in the abundance and overall diversity of the microbiota across the three groups (Fig 6C-6F). Furthermore, a Principal Coordinates Analysis (PCoA) of β diversity indicated that the microbial community structures of the NC group and the PLGC+H-WFC group were closely aligned, while a distinct divergence was evident in the microbial community structure of the PLGC group compared to the other two groups (Fig 6G-6I). Therefore, H-WFC may play a role in regulating gastric microbial homeostasis.

The Veen graph showed that there were 684 bacterial genera that were commonly enriched in the NC group, PLGC group, and PLGC+H-WFC group (Fig 6J). The analysis focused on quantifying the abundance of ASV/OTU at the genus level in each respective area. Notably, the genus *Coprococcus* exhibited significantly higher abundance levels in both the NC group and the PLGC+H-WFC group compared to the PLGC group (Fig 6K). The species composition analysis, utilizing a heatmap for UPGMA cluster analysis, revealed a high abundance of *Turicibacter*, *Butyricicoccus*, *Treponema*, and *Coprococcus* in both the NC and PLGC+H-WFC groups, while these genera exhibited markedly low abundance in the PLGC group, suggesting that these bacteria may be related to the effects of WFC (Fig 6L).

**Fig 7:**
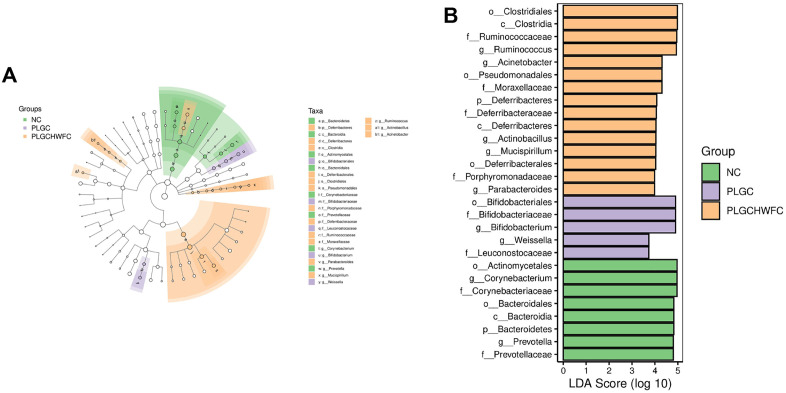
The differences of gastric contents among groups. **(A)** Cladogram of LEfSe analysis. **(B)** Histogram of LEfSe analysis.

Moreover, the LEfSe method was used to find biomarkers in the gastric contents among NC, PLGC and LGC + H-WFC groups. The NC group were significantly enriched for *o_Actinomycetales*, *g_Corynebacterium* and *f_Corynebacteriaceae*, the PLGC group were significantly enriched for *o_Bifidobacteriales*, *f_Bifidobacteriaceae* and *g_Bifidobacterium*, the LGC + H-WFC group were significantly enriched for *o_Clostridiales*, *c_Clostridia*, *f_Ruminococcaceae* and *g_Ruminococcus* (Fig 7).

### 16S rRNA sequencing analysis of rat intestinal microbiota

**Fig 8:**
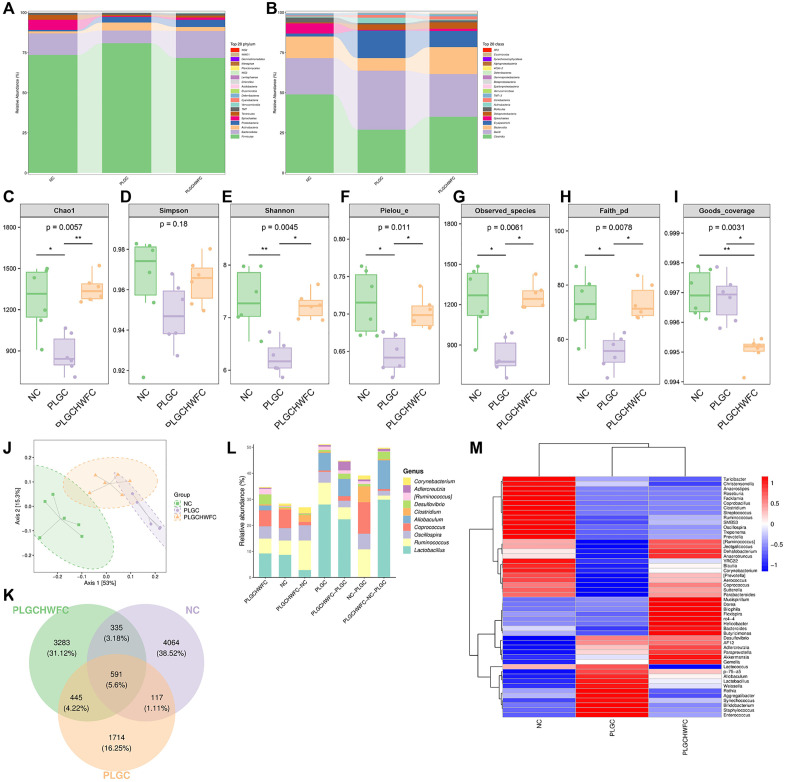
16SrRNA sequencing analysis of rat intestinal microbiota. (A) Species enrichment at the phylum level; (B) Species enrichment at the genus level; (C) Analysis of microbial α-diversity index; (D) Analysis of microbial β-diversity index; (E) Venn diagram of sample ASV/OUT; (F) ASV/OTU abundance histograms in different regions of the Venn diagram at the genus level; (G) Heat map of species composition at the genus level with species clustering. n = 6. *P < 0.05, **P < 0.01.

It is widely recognized that there is a significant interconnection among the various components of the gastrointestinal tract. To further investigate the influence of WFC on intestinal microbiota, we performed sequencing of the intestinal microbiota in rats utilizing 16S rRNA gene analysis. The sequencing results showed that the dominant bacteria in the rat intestine. The top three species in relative abundance at the phylum level were *Firmicutes*, *Bacteroides* and *Actinobateria* (Fig 8A), and the top three species in relative abundance at the class level were *Clostridia*, *Bacilli* and *Bacteroidia* (Fig 8B). Sample diversity of rat intestinal microbiota was analyzed by microbial alpha diversity (Fig 8C-8I). The abundance, diversity and uniformity of intestinal microbiota in rats in the NC group and PLGC+H-WFC group were significantly higher than those in the PLGC group. There were no significant differences between the NC and PLGC+H-WFC groups except for the Goods_coverage index. Comparing β diversity through principal coordinate analysis (PCoA), it was found that there were significant differences in microbial community structure between the NC group and the PLGC group, but the microbial community structure of the PLGC+H-WFC group was similar to both groups (Fig 8J).

The abundance of ASV/OTU in intestinal microbiota was analyzed by the Veen diagram (Fig 8K). The results showed that similar to the results of gastric content analysis, the abundance of *Coprococcus* in the NC group and PLGC+H-WFC group was significantly higher than that in PLGC (Fig 8L). We performed a species composition analysis utilizing UPGMA cluster analysis heatmaps. The results revealed that the abundance of the bacterial strains *Jeotgalicoccus*, *Dehalobacterium*, *Anaerotruncus*, *Coprococcus*, *Sutterella*, and *Parabacteroides* was significantly higher in both the NC group and the PLGC+H-WFC group. In contrast, these strains exhibited markedly low abundance in the PLGC group (Fig 8M).

**Fig 9:**
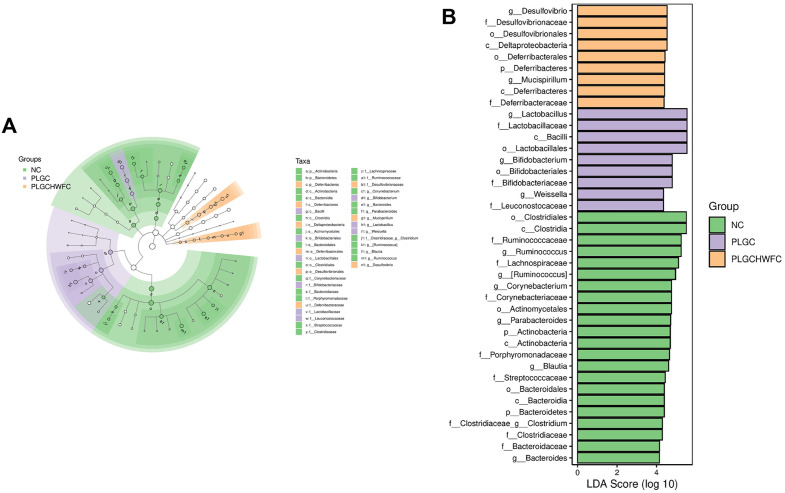
The differences in intestinal microbiota among groups. (A) Cladogram of LEfSe analysis. (B) Histogram of LEfSe analysis.

In addition, LEfSe was applied to find biomarkers in the intestinal microbiota among NC, PLGC and LGC + H-WFC groups. The NC group were significantly enriched for *o_Clostridiales*, *c_Clostridia*, *f_Ruminococcaceae* and *g_Ruminococcus*, the PLGC group were significantly enriched for *g_Lactobacillus*, *f_Lactobacillaceae*, *c_Bacill* and *o_Lactobacillales*, the LGC + H-WFC group were significantly enriched for *g_Desulfovibrio*, *f_Desulfovibrionaceae*, *o_Desulfovibrionales* and *c_Deltaproteobacteria* (Fig 9).

### Screening for key bacteria by combined analysis of 16S rRNA

**Fig 10:**
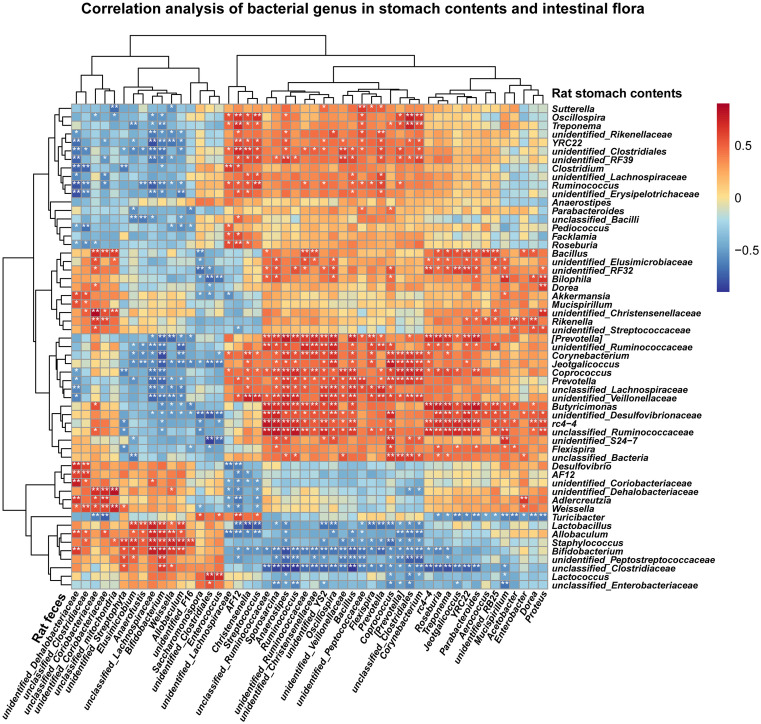
Correlation analysis of bacterial genus in gastric contents and intestinal microbiota. Correlation analysis was conducted using Spearman’s method for statistical analysis. Bioinformatic analysis was performed using the OmicStudio tools at https://www.omicstudio.cn/tool. ^*^P < 0.05, ^**^P < 0.01.

To further investigate the interaction between gastric microbiota and fecal microbiota, as well as their correlation with WFC’s improvement of PLGC, we undertook a comprehensive correlation analysis between the microbial communities present in gastric content and those in the intestinal microbiota. This analysis was visually represented through a heat map (Fig 10), which facilitated the identification of significant correlations. The results revealed notable associations between various bacterial genera, including *Allobaculum*, *Bifidobacterium*, *Ruminococcus*, *Coprococcus*, and *Corynebacterium*, among others.

**Fig 11:**
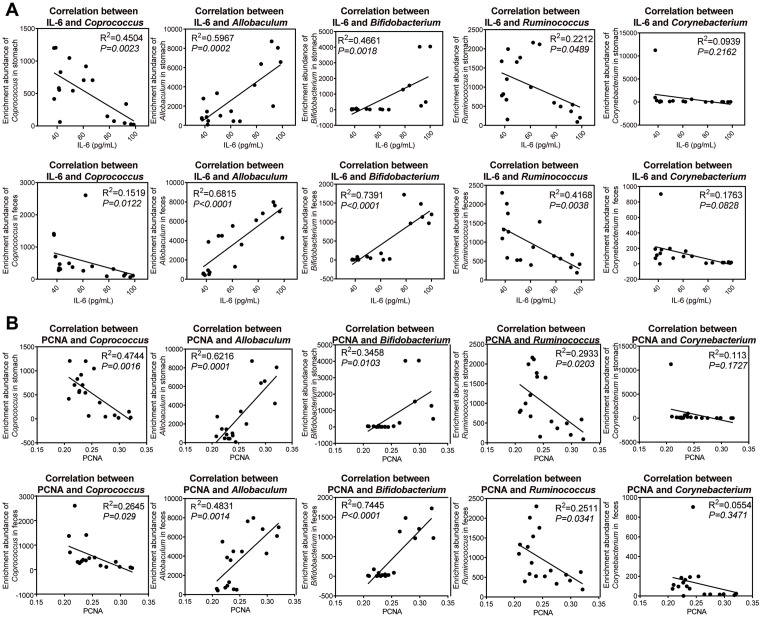
Correlation analysis of gastrointestinal microbiota with IL-6 and PCNA. **(A)** Correlation analysis of gastrointestinal microbiota with IL-6; **(B)** Correlation analysis of gastrointestinal microbiota with PCNA. Correlation analysis was conducted using Spearman’s method for statistical analysis. *n* = 6. ^*^P < 0.05, ^**^P < 0.01.

To explore the correlation between the above bacterial genera and the IL-6 pathway in gastric microorganisms and intestinal microorganisms, we conducted a correlation analysis. As shown in Fig 11A, *Coprococcus* and *Ruminococcus* were significantly negatively correlated with IL-6 levels in the gastrointestinal microbiota, while *Allobaculum* and *Bifidobacterium* were significantly positively correlated with IL-6 levels. We also performed a correlation analysis between the aforementioned bacterial genera and the CAG cell proliferation index PCNA. As illustrated in Fig 11B, *Coprococcus* and *Ruminococcus* exhibited a significant negative correlation with CAG cell proliferation, whereas *Allobaculum* and *Bifidobacterium* demonstrated a significant positive correlation with CAG cell proliferation.

## Discussion

CAG is a prevalent and chronic digestive disorder characterized by the infiltration of inflammatory cells and the atrophy of gastric mucosal glands, primarily resulting in a reduction or complete loss of parietal cells [40]. This condition is significant in the context of the Correa cascade, wherein CAG can progress to metaplasia and dysplasia, ultimately increasing the risk of gastric cancer [10]. WFC is a traditional Chinese herbal compound that is widely used in clinical practice to treat CAG. Although WFC has been reported to be more effective than Western medicine and other Chinese patent medicine in improving gastric mucosal under gastroscopy, improving histopathologic changes of gastric mucosa, and inhibiting Helicobacter pylori, and no significant difference in safety was examined between WFC and aforementioned drugs, the role and mechanism of WFC in the gastrointestinal microbiota of CAG is still unclear [41]. This study is the first to find that WFC alleviates MNNG-induced CAG by improving the gastrointestinal microbiota homeostasis and inhibiting the IL-6/JAK1/STAT3 pathway. Furthermore, this study clarifies WFC’s regulatory effects on gastric microbiota, and reveals the interaction between gastrointestinal microbiota and the IL-6/JAK1/STAT3 pathway in CAG progression, thereby providing novel insights into WFC’s multi-targeted therapeutic mechanism on CAG.

Animal models play an essential role in the drug development of treatments for human diseases. CAG rodent models can be established through various methods, including chemical stimuli such as ethanol, ammonia water and sodium deoxycholate, as well as Helicobacter pylori infection, autoimmunity against gastric mucosa homogenization and MNNG exposure [42]. Notably, the MNNG-induced method is commonly employed for creating CAG models, due to MNNG-induced animal models displaying pathological characteristics that closely resemble human CAG [43]. In this study, the rats in the model group exhibited significant loss of gastric mucosal epithelial cells, pronounced atrophy of the glands and a marked infiltration of inflammatory cells, indicating the CAG animal model was successfully established.

Recent studies indicate that nucleosides, flavones, organic acids, and phenylpropanoids play significant roles in gastric diseases. For example, ganciclovir, a nucleoside analogue, effectively halts the progression of gastrointestinal disorders [44]. Luteolin, a flavone ingredient, targets the AGE-RAGE signaling to reduce inflammation and ferroptosis in CAG [45]. In addition, both organic acids and stomach acids are crucial in modulating the gut microbiome [46]. Additionally, researchers have identified 63 compounds, including 5 phenylpropanoids, in dried ginger, which is frequently used for the prevention and treatment of gastrointestinal tract-related diseases [47]. Consistent with previous research, this study found the majority of the compounds identified from WFC were nucleosides, flavones, organic acids, phenylpropanoids and other components. Thus, we hypothesized that WFC may possess the potential to alleviate CAG.

IL-6 is a multifunctional proinflammatory cytokine synthesized by various cell types, and it serves a crucial role in the mediation of inflammatory processes, and the promotion of tumorigenesis [48–50]. The IL-6/JAK1/STAT3 signal axis is implicated in promoting inflammatory responses and cellular proliferation [51,52], thereby influencing the tumor microenvironment and potentially exacerbating the susceptibility to gastric carcinogenesis [53,54]. In this study, the IL-6 level in the PLGC group was significantly higher than that in the NC group, and WFC treatment effectively inhibited IL-6, and its therapeutic effect was dose-dependent on WFC concentration. WFC also inhibited the JAK1/STAT3 pathway. Meanwhile, we observed that WFC treatment effectively reduced the cell proliferation index PCNA, suggesting that WFC may regulate IL-6 to inhibit PLGC inflammation levels and cell proliferation.

The activation of inflammatory response, disruption of gut microbiota, and changes in fecal metabolic phenotype may be closely related to the occurrence of PLGC [55–57]. Therefore, this study started from the gastric content microorganisms and intestinal microbiota and explored the potential mechanism of action of WFC in the treatment of CAG in PLGA through 16S rRNA technology analysis. Interestingly, in the two sequencing results, *Coprococcus* was highly abundant in the NC group rats and the PLGC+H-WFC group rats, but very low in the PLGC group, suggesting that *Coprococcus* may be an important bacterial species for WFC to improve PLGC.

*Coprococcus* is a significant genus within the Firmicutes family, specifically the *Lachnospiraceae*, and plays a vital role in the composition of intestinal microbiota [58]. Predominantly isolated from fecal matter, species of *Coprococcus* are known for their active fermentation of carbohydrates and their role as key producers of *butyrate*, a short-chain fatty acid crucial for gut health, akin to the contributions of Faecalibacterium prausnitzii [59]. Recent research has indicated that *Coprococcus* may serve as an important microbial biomarker for assessing the health of the human gastrointestinal tract [6,60,61]. The relationship between *Coprococcus* and IL-6 may involve the regulation of intestinal health and inflammatory response [62,63]. *Coprococcus* may exert its probiotic effects by affecting IL-6 levels, although the specific mechanisms may vary among different hosts and disease models [64–66]. For example, one study showed that *Coprococcus* could alleviate colon inflammation in mice, and this protective effect may be related to its reduced expression of inflammatory cytokines [58]. Research suggests that the active ingredient Renshen in WFC can effectively mitigate colitis by reducing cell infiltration and lowering levels of inflammatory markers such as IL-6 and TNF-α [67]. Additionally, it regulates gut microbiota diversity and composition, decreases the relative abundance of *Coprococcus*, and supports the recovery of both gut microbiota and the intestinal mucosal barrier [68]. This suggests a potential mechanism by which WFC acts on PLGC inflammation by regulating gut microbiota homeostasis.

In this study, we identified a significant negative linear relationship between *Coprococcus* levels and the inflammatory cytokine IL-6, alongside the cellular proliferation marker PCNA, through comprehensive correlation analysis. These findings indicate that the presence of WFC may play a crucial role in modulating IL-6 levels by sustaining the homeostasis of *Coprococcus* and promoting the synthesis of butyrate. This mechanism appears to impede the IL-6/JAK1/STAT3 signaling pathway, ultimately contributing to the inhibition of CAG cell proliferation. This suggests a potential therapeutic avenue for the regulation of inflammatory responses and cancer cell growth in related contexts.

This study offers compelling scientific evidence for the role of gastric and intestinal microbiota in the onset and progression of PLGC. Nevertheless, the study had some limitations. Firstly, the study primarily relied on the classic MNNG-induced PLGC animal models. Although animal models are effective at simulating key pathologies, they cannot fully replicate human physiological complexity, which limits the generalizability of the findings of the study. Secondly, the small sample size was another limitation of the study. Thirdly, while we identified the main chemical components of WFC via UPLC-Q/TOF-MS, quantitative analysis of specific marker compounds was not conducted. In addition, there was a risk of overinterpreting the results of the microbiota analysis. Furthermore, this study primarily focused on characterizing the correlation between *Coprococcus* abundance and key pathological indicators (IL-6, PCNA), but did not perform in-depth functional prediction for *Coprococcus* role in microbial metabolism or immune-related pathways. To enhance our understanding of the influence of WFC on gastrointestinal microbiota homeostasis, further clinical observations and more samples are necessary. Additionally, methods for accurately quantifying key active components in WFC should be supplemented, and fecal microbiota transplantation should be used to further investigate the key results of the study, thereby making the results of the study more convincing. In addition, functional prediction analyses (such as PICRUSt2) should be supplemented to systematically explore the metabolic capabilities (e.g., butyrate synthesis) of *Coprococcus* and its regulatory effects on host immune pathways, thereby providing more robust evidence for its role in the therapeutic mechanism of WFC against PLGC.

## Conclusion

In recent discussions about the occurrence and development of PLGC, it is widely acknowledged that the balance of gastric and intestinal microbiota plays a crucial role. However, the interactions and underlying mechanisms remain inadequately supported by scientific evidence. This study utilized 16S rRNA sequencing technology to analyze the microbiota in both the stomach and intestines, providing insights into potential interactions that may contribute to the progression of PLGC. Additionally, while WFC (a candidate therapeutic agent) shows promise, the lack of mechanistic research has hindered its clinical application. This research aims to fill that gap by offering scientific evidence regarding WFC’s mechanisms, suggesting that it may maintain the homeostasis of *Coprococcus*, which in turn lowers IL-6 levels and inhibits the JAK1/STAT3 signaling pathway. This inhibition could reduce inflammation and the proliferation of CAG cells, potentially obstructing the malignant phenotype associated with the disease.

## Supporting information

S1 Table Chromatographic conditions and mass spectrum conditions. (PDF)

S2 Table The compounds of WFC identified in positive ion mode. (PDF)

S3 Table The compounds of WFC identified in negative ion mode. (PDF)
