# Supplementary material for: Weifuchun alleviates MNNG-induced chronic atrophic gastritis by improving the gastric and intestinal microbiota homeostasis
Source: PLoS One. 2025 Nov 24;20(11):e0333375. doi: 10.1371/journal.pone.0333375 (PMC12643289; doi:10.1371/journal.pone.0333375)
Supplement: S1 Table — (PDF) [file pone.0333375.s001.pdf]

Supplementary Table 1 Chromatographic conditions and mass spectrum conditions

Column type	ACQUITY UPLC HSS T3, 1.8 μm, 2.1 mm×100 mm
Flow rate	0.4 mL/min
A phase	0.1% formic acid in water
B phase	0.1% formic acid in acetonitrile
Time, (% B)	0 min-17.5 min, 3%-90%
Time, (% B)	17.5 min-21 min, 90%
Time, (% B)	21 min-22 min, 90%-3%
Time, (% B)	22 min-25 min, 3%
Column temperature	40 °C
Injection volume	5 μL
Time of flight mass spectrometer	SCIEX QTOF 6600
Ion Source Gas1	55
Ion Source Gas2	55
Curtain gas	35
Source temperature	550 °C
IonSapary Voltage Floating	5500 V/-4500 V(positive and negative ion mode)
TOF-MS scan m/z range	25-1000 Da
Production scan m/z range	25-1500 Da
TOF-MS scan accumulation time	0.25 s/spectra
Product ion scan accumulation time	0.035 s/spectra
Declustering potential	+60 V (positive and negative ion mode)
Collision Energy	30±15 eV
Exclude isotopes	within 4 Da
Candidate ions to monitor per cycle	15
