# Supplementary material for: Weifuchun alleviates MNNG-induced chronic atrophic gastritis by improving the gastric and intestinal microbiota homeostasis
Source: PLoS One. 2025 Nov 24;20(11):e0333375. doi: 10.1371/journal.pone.0333375 (PMC12643289; doi:10.1371/journal.pone.0333375)
Supplement: S2 Table — (PDF) [file pone.0333375.s002.pdf]

Supplementary Table 2 The compounds of WFC identified in positive ion mode

Name	Formula	Mass (Da)	m/z (Da)	RT (min)	Library Score	Area	Error (ppm)
L-Arginine	C6H14N4O2	174.1117	175.1190	0.74	89%	21804	0.46
Stachydrine	C7H13NO2	143.0946	144.1025	0.77	89%	504207	4.16
N-Acetylornithine	C7H14N2O3	174.1004	175.1079	0.77	76%	21229	0.72
Cytidine	C9H13N3O5	243.0855	244.0942	1.06	100%	6181	5.92
Adenine	C5H5N5	135.0545	136.0624	1.06	95%	38594	4.86
4-Dimethylaminobenzaldehyde	C9H11NO	149.0841	150.0919	1.07	77%	84722	3.57
Phenylephrine	C9H13NO2	167.0946	168.1018	1.07	82%	6387	-0.85
Nicotinic acid	C6H5NO2	123.0320	124.0397	1.08	99%	12474	3.16
Niacinamide	C6H6N2O	122.0480	123.0554	1.13	99%	13490	0.98
Citric acid	C6H8O7	192.0270	193.0342	1.14	68%	2420	-0.62
Hypoxanthine	C5H4N4O	136.0385	137.0458	1.17	90%	2006	-0.29
Pyridoxine	C8H11NO3	169.0739	170.0813	1.17	95%	3898	0.53
Adenosine monophosphate	C10H14N5O7P	347.0631	348.0722	1.19	98%	6218	5.35
Guanosine monophosphate	C10H14N5O8P	363.0580	364.0656	1.27	100%	1477	0.82
N-Acetylglutamic acid	C7H11NO5	189.0637	190.0710	1.32	99%	5949	0.21
Uridine	C9H12N2O6	244.0695	245.0769	1.34	98%	3709	0.56
2-Pyrrolidinone	C4H7NO	85.0528	86.0602	2.07	63%	25842	1.83
Adenosine	C10H13N5O4	267.0967	268.1058	2.08	100%	88758	6.78
Cordycepin	C10H13N5O3	251.1018	252.1098	2.15	100%	2333	2.73
Guanosine	C10H13N5O5	283.0917	284.1011	2.15	100%	14172	7.59

Hordenine	C10H15NO	165.1154	166.1226	2.20	96%	855	0.00
3-Hexenedioic acid	C6H8O4	144.0423	145.0497	2.24	75%	17082	1.40
Thr	C4H9NO3	119.0582	120.0811	2.45	99%	224	129.72
Pantothenic acid	C9H17NO5	219.1107	220.1181	2.68	92%	7943	0.66
Indoleacrylic acid	C11H9NO2	187.0633	188.0715	3.20	95%	3375	4.56
L-Tryptophan	C11H12N2O2	204.0899	205.0972	3.20	99%	3392	0.35
Salidroside +NH3	C14H20O7.NH3	317.1475	318.1550	3.28	84%	1937	0.93
Codeine	C18H21NO3	299.1521	300.1594	3.36	87%	6354	-0.04
Esculin hydrate	C15H16O9	340.0794	341.0865	3.43	94%	3346	-0.66
Chlorogenic acid	C16H18O9	354.0951	355.1023	3.81	99%	5985	-0.13
Quercetin 3-O-sophoroside	C27H30O17	626.1483	627.1555	3.83	94%	2001	-0.07
Syringin +NH3	C17H24O9.NH3	389.1686	390.1751	3.97	98%	2494	-1.98
Pseudoginsenoside-RT5	C36H62O10	654.4343	655.4410	4.19	81%	485	-0.99
Vitamin B2	C17H20N4O6	376.1383	377.1468	4.35	100%	6481	3.18
Hydrocinnamic acid	C9H10O2	150.0681	151.0753	4.62	62%	725	-0.70
Methyl 2-hydroxybenzoate	C8H8O3	152.0473	153.0548	4.65	87%	4333	0.88
Schaftoside	C26H28O14	564.1479	565.1550	4.73	96%	3788	-0.22
Eleutheroside E +NH3	C34H46O18.NH3	759.2950	760.2959	4.85	69%	707	-8.34
Orientin	C21H20O11	448.1006	449.1115	4.91	98%	2308	8.22
Rutin	C27H30O16	610.1534	611.1614	5.26	97%	20026	1.15
Vitexin	C21H20O10	432.1057	433.1132	5.31	99%	5656	0.53
Eriodictyol	C15H12O6	288.0634	289.0713	5.34	96%	7867	2.08

Isoscopoletin	C10H8O4	192.0423	193.0494	5.45	99%	3508	-0.65
Quercetin	C15H10O7	302.0427	303.0500	5.47	94%	15395	0.07
Hyperin	C21H20O12	464.0955	465.1028	5.47	100%	10496	-0.02
Luteolin-7-O-beta-D-glucuronide	C21H18O12	462.0798	463.0875	5.53	92%	3864	0.86
Apigenin	C15H10O5	270.0528	271.0621	5.62	99%	4761	7.55
Protopine	C20H19NO5	353.1263	354.1338	5.88	78%	873	0.52
Naringin	C27H32O14	580.1792	581.1867	5.91	86%	111622	0.36
Naringenin	C15H12O5	272.0685	273.0762	5.91	100%	135794	1.77
Rhoifolin	C27H30O14	578.1636	579.1708	5.98	99%	33123	-0.16
Apigenin 7-O-beta-D-glucuronide	C21H18O11	446.0849	447.0925	6.15	100%	4054	0.64
Vitamin A acid	C20H28O2	300.2089	301.2163	6.15	66%	4516	0.34
Hesperetin	C16H14O6	302.0790	303.0878	6.26	99%	141411	4.87
Hesperidin	C28H34O15	610.1898	611.1976	6.27	92%	136851	0.98
Nodakenin	C20H24O9	408.1420	409.1495	6.33	95%	2216	0.36
Tuberostemonine	C22H33NO4	375.2410	376.2488	7.13	79%	15823	1.57
Linarin	C28H32O14	592.1792	593.1874	7.26	100%	5881	1.56
Pinocembrin	C15H12O4	256.0735	257.0809	7.41	93%	868	0.42
Isosakuranetin	C16H14O5	286.0841	287.0917	7.53	61%	50264	1.01
Tectorigenin	C16H12O6	300.0634	301.0716	7.80	94%	11067	3.04
Kirenol +NH3	C20H34O4.NH3	355.2722	356.2794	8.22	66%	893	-0.42
Psoralen	C11H6O3	186.0317	187.0393	8.69	92%	3495	1.74

Uvaol	C30H50O2	442.3811	443.3887	8.77	69%	2890	0.70
Pulegone	C10H16O	152.1201	153.1279	9.20	83%	29467	3.35
Isopimpinellin	C13H10O5	246.0528	247.0603	9.48	90%	748	0.94
Xanthotoxol	C11H6O4	202.0266	203.0336	9.51	92%	818	-1.22
Gypenoside XVII	C48H82O18	946.5501	947.5598	9.69	84%	786	2.56
Limonin	C26H30O8	470.1941	471.2027	10.07	98%	102711	2.90
Nobiletin	C21H22O8	402.1315	403.1403	10.71	93%	1808043	3.79
Panaxydol	C17H24O2	260.1776	261.1850	11.57	88%	7105	0.30
Obacunone	C26H30O7	454.1992	455.2067	11.58	85%	46361	0.51
Asiatic acid	C30H48O5	488.3502	489.3564	12.14	65%	1416	-2.13
Momordin Ic +NH3	C41H64O13.NH3	781.4612	782.4629	12.23	68%	237	-7.14
Osthole	C15H16O3	244.1099	245.1179	12.62	95%	5548	2.72
Kaurenoic acid	C20H30O2	302.2246	303.2322	13.56	70%	3574	0.97
Corosolic acid	C30H48O4	472.3553	473.3618	14.44	86%	856	-1.51
Betulonicacid	C30H46O3	454.3447	455.3514	14.45	85%	1381	-1.34
