# Supplementary material for: Weifuchun alleviates MNNG-induced chronic atrophic gastritis by improving the gastric and intestinal microbiota homeostasis
Source: PLoS One. 2025 Nov 24;20(11):e0333375. doi: 10.1371/journal.pone.0333375 (PMC12643289; doi:10.1371/journal.pone.0333375)
Supplement: S3 Table — (PDF) [file pone.0333375.s003.pdf]

Supplementary Table 3 The compounds of WFC identified in negative ion mode

Name	Formula	Mass (Da)	m/z (Da)	RT (min)	Library Score	Area	Error (ppm)
L-Malic acid	C4H6O5	134.0215	133.0150	0.64	97%	276958	5.87
Quinic acid	C7H12O6	192.0634	191.0560	0.65	97%	507950	-0.33
Glyceric acid	C3H6O4	106.0266	105.0195	0.66	85%	15937	1.67
Malonic acid	C3H4O4	104.0110	103.0035	0.70	84%	19455	-1.93
Pyroglutamic acid	C5H7NO3	129.0426	128.0352	0.71	81%	120409	-0.57
N-Acetylglutamic acid	C7H11NO5	189.0637	188.0566	0.71	93%	31331	0.81
Turanose	C12H22O11	342.1162	341.1086	0.74	90%	263148	-0.98
N-Acetylornithine	C7H14N2O3	174.1004	173.0927	0.75	92%	84628	-2.80
Pipecolic acid	C6H11NO2	129.0790	128.0716	0.76	81%	6052	-0.84
Succinic acid	C4H6O4	118.0266	117.0192	0.76	99%	195692	-0.94
3-Hydroxybutyric acid	C4H8O3	104.0473	103.0401	0.81	62%	2135	0.67
Ferulic Acid	C10H10O4	194.0579	193.0507	0.96	93%	151473	0.55
Gallic acid	C7H6O5	170.0215	169.0142	0.96	91%	5736	-0.23
Caffeic acid	C9H8O4	180.0423	179.0358	0.98	87%	140644	4.48
Danshensu	C9H10O5	198.0528	197.0466	0.99	97%	524871	5.23
p-Coumaric acid	C9H8O3	164.0473	163.0400	1.06	87%	18940	-0.56
2-Isopropylmalic acid	C7H12O5	176.0685	175.0611	1.12	95%	58815	-0.63
Hydroxyphenyllactic acid	C9H10O4	182.0579	181.0508	1.14	68%	18240	0.66
2,3-Dihydroxybenzoic acid	C7H6O4	154.0266	153.0196	1.16	98%	53864	1.41
Cichoric acid	C22H18O12	474.0798	473.0730	1.19	91%	9807	0.84

Pantothenic acid	C9H17NO5	219.1107	218.1040	1.21	92%	11628	2.65
Adenosine 2",3"-cyclic phosphate	C10H12N5O6P	329.0525	328.0466	1.23	97%	8469	4.20
4-Pyridoxic acid	C8H9NO4	183.0532	182.0459	1.59	92%	3863	-0.12
Cryptochlorogenic acid	C16H18O9	354.0951	353.0877	1.92	98%	75812	-0.23
Guanosine	C10H13N5O5	283.0917	282.0843	1.98	99%	24049	-0.27
Adenine	C5H5N5	135.0545	134.0467	1.99	94%	22789	-3.71
L-Tryptophan	C11H12N2O2	204.0899	203.0826	2.91	93%	1649	0.18
Hydroxytyrosol	C8H10O3	154.0630	153.0557	2.92	94%	8702	-0.34
Rosmarinic acid	C18H16O8	360.0845	359.0770	3.14	100%	2318240	-0.56
Salidroide	C14H20O7	300.1209	299.1133	3.30	76%	1193	-1.14
Esculin hydrate	C15H16O9	340.0794	339.0721	3.32	74%	4944	-0.06
Scutellarin	C21H18O12	462.0798	461.0725	3.36	100%	4827	-0.11
Protocatechuic Aldehyde	C7H6O3	138.0317	137.0245	3.50	96%	68333	0.36
Azelaic acid	C9H16O4	188.1049	187.0975	3.62	77%	29363	-0.52
Isochlorogenic acid C	C25H24O12	516.1268	515.1195	3.79	100%	18576	0.01
Esculetin	C9H6O4	178.0266	177.0187	4.10	96%	18376	-3.69
Vicenin-II	C27H30O15	594.1584	593.1515	4.26	92%	103471	0.47
Rutin	C27H30O16	610.1534	609.1461	5.28	99%	59430	-0.03
Vitexin	C21H20O10	432.1057	431.0978	5.29	100%	4371	-1.22
Neoeriocitrin	C27H32O15	596.1741	595.1686	5.40	97%	90233	2.90
Verbascoside	C29H36O15	624.2054	623.1990	5.48	98%	1235	1.34
Hyperin	C21H20O12	464.0955	463.0884	5.49	100%	35299	0.48

Luteoloside	C21H20O11	448.1006	447.0937	5.57	100%	2746	0.88
Traumatic acid	C12H20O4	228.1362	227.1290	5.71	87%	13186	0.67
Narirutin	C27H32O14	580.1792	579.1723	5.81	92%	187239	0.59
Neohesperidin	C28H34O15	610.1898	609.1827	6.32	99%	179766	0.40
Ginsenoside-Ro	C48H76O19	956.4981	955.4965	6.66	98%	25126	5.97
Ginsenoside Rg1 +HCOOH	C42H72O14.HCOOH	846.4977	845.4801	6.89	92%	891	-12.24
Chikusetsusponin IVa	C42H66O14	794.4453	793.4394	7.07	100%	1725	1.77
Luteolin	C15H10O6	286.0477	285.0406	7.51	96%	5264	0.65
Quercetin	C15H10O7	302.0427	301.0355	7.62	85%	1612	0.24
Syringaresinol	C22H26O8	418.1628	417.1555	7.68	74%	1290	-0.02
Caffeic acid ethyl ester	C11H12O4	208.0736	207.0666	7.82	95%	6247	1.32
12-Hydroxydodecanoic acid	C12H24O3	216.1725	215.1655	8.11	100%	2240	0.92
Naringenin	C15H12O5	272.0685	271.0615	8.23	93%	34879	1.13
Apigenin	C15H10O5	270.0528	269.0436	8.40	85%	4923	-7.40
Hesperetin	C16H14O6	302.0790	301.0715	8.60	91%	14438	-0.86
Aurantio-Obtusin	C17H14O7	330.0739	329.0667	8.84	73%	99698	0.09
Ginsenoside Rc	C53H90O22	1078.5924	1077.5881	9.03	71%	4810	2.78
7-Hydroxycoumarin	C9H6O3	162.0317	161.0235	9.24	97%	38610	-5.68
Ginsenoside F1 +HCOOH	C36H62O9.HCOOH	684.4449	683.4357	9.30	74%	1296	-2.78
Diosmetin	C16H12O6	300.0634	299.0563	9.71	97%	11860	0.61

Gypenoside XVII	C48H82O18	946.5501	945.5437	9.71	75%	9049	0.93
Ginsenoside Rd +HCOOH	C48H82O18.HCOOH	992.5556	991.5476	9.72	99%	597	-0.74
7-Demethylsuberosin	C14H14O3	230.0943	229.0875	9.95	67%	55745	1.96
Ladanein	C17H14O6	314.0790	313.0715	10.19	97%	40384	-0.75
Notoginsenoside Ft1	C47H80O17	916.5396	915.5327	10.47	97%	357	0.47
Eupatilin	C18H16O7	344.0896	343.0822	10.50	87%	21194	-0.26
Genkwanin	C16H12O5	284.0685	283.0609	10.79	81%	936	-0.99
Asiatic acid	C30H48O5	488.3502	487.3430	11.75	89%	34991	0.26
20(R)-Ginsenoside Rg3	C42H72O13	784.4973	783.4897	11.92	100%	13174	-0.47
Gingerglycolipid B +HCOOH	C33H58O14.HCOOH	724.3881	723.3826	13.08	97%	224	2.38
Corosolic acid	C30H48O4	472.3553	471.3475	14.32	100%	18049	-0.97
Oleanolic acid	C30H48O3	456.3604	455.3535	17.19	100%	9212	0.94
Linoleic acid	C18H32O2	280.2402	279.2330	17.49	100%	65630	0.03
Elaidic acid	C18H34O2	282.2559	281.2485	18.63	100%	3318	-0.47
Stearic acid	C18H36O2	284.2715	283.2642	20.16	80%	7588	-0.22
